# Dying for a Tan: A Survey to Assess Solarium Adherence to World Health Organization Guidelines in Australia, New Zealand, and the United Kingdom

**Published:** 2013-12-26

**Authors:** Amali Chandrasena, Kavit Amin, Barry Powell

**Affiliations:** St. Georges Hospital, London, the United Kingdom

**Keywords:** solarium/sunbed, melanoma, skin cancer, tan, Ultraviolet

## Abstract

**Background:** Increased use of solariums worldwide has led to health concerns linking regular exposure to ultraviolet light with skin cancer. Most studies have attempted to delineate attitudes and motivations of users, with little inquiry into those entrusted as “service providers.” **Methods:** A questionnaire was designed specifically for the survey on the basis of World Health Organization guidelines. Twenty-one solariums in major cities from each country were randomly chosen via telephone directory and visited in Australia, New Zealand, and the United Kingdom. A prospective customer asked a series of questions to assess conformity with these guidelines. **Results:** Solariums in Australia were most successful at adhering to World Health Organization guidelines, followed closely by those in the United Kingdom. Overall, solariums in New Zealand did not comply as well, especially with the use of consent forms, and did not adequately inform customers of the potential adverse side effects including skin cancer risks. **Conclusions:** Our data have shown several differences in the way solariums conform to guidelines in our 3 chosen countries. A plausible reason for these differences is the difference in solarium regulations worldwide. The limited number of solariums selected for the survey, together with individual country variation, does not enable firm conclusions to be drawn about the operations of solariums in each country. However, it does provide knowledge of the variation between solariums and further suggests the need to evaluate the workings of these service providers in each country.

It is known that increased levels of acute exposure to the sun result in a suntan. Furthermore, intense exposure to ultraviolet (UV) light can damage skin cells leading to sunburn and eventually skin cancer.^1^ The link between sunbed use and skin cancer has become a very topical concern worldwide. The Cancer Research UK Policy statement highlights the increasing evidence linking sunbeds to an elevated risk of cancer, most notably, malignant melanoma.^2^ Malignant melanoma is the most aggressive form of skin cancer and is the cause of many preventable deaths. Those at highest risk possess fair skin, an increased number of moles, have a positive family history, and have been exposed to increased measures of UV radiation.^3^ Recent research claims that sunbed exposure may significantly increase the risk of malignant melanoma in individuals who find it difficult to tan.^4^ The idea that sunbeds pose adverse health risks has been documented in the 1994 major scientific review carried out by the World Health Organization (WHO).^5^

The world incidence rate of malignant melanoma among white populations in high-risk areas is highest in Australia and New Zealand.^6^ Between 1996 and 2006, Australia has seen a 200% growth of the tanning industry in Adelaide and Brisbane, with a further 500% in Melbourne and 1000% in Perth.^7^ A New Zealand audit documented a 241% increase in tanning salons from the *Yellow Pages* directory between 1992 and 2006. There was also a 525% increase in wholesale trade, signifying the ever-powerful expansion of the industry.^8^ New Zealand, Australia, and the United Kingdom are a few countries that have been experiencing media attention for the potential problems tanning salons pose. The Australian government has taken account for this and has changed legislation from a voluntary code of practice to mandatory practice in 2008. Regulations are on a state-by-state basis and include a ban on the use of solariums for those younger than 18 years.^8^ Current legislation states:

- Any person younger than 18 years is not allowed to use a solarium.
- Any person with skin type 1 is not allowed to use a solarium.
- Every client has a skin-type assessment conducted before using a solarium.
- Every client signs the prescribed consent form before using a solarium.
- Proof-of-age documents are sighted prior to a client signing a consent form.
- Mandatory health warnings are displayed.

New Zealand is also aware of this public health problem, whereby key individuals have called for the New Zealand government to follow Australia's mandatory guidelines. The United Kingdom currently conforms to the Sunbed Regulation Act 2010. At present, its main focus is upon ensuring an age restriction of 18 years, not specifying skin type, assessment, or mandatory health warnings. One UK study found that over quarter of a million children aged between 11 and 17 years used tanning salons regularly.^9^ Despite the notion that 95% of cases are largely preventable by decreasing UV exposure, the belief that a tan is considered cosmetically pleasing is universally accepted.^10^ One study examined children's attitudes and behaviors toward indoor tanning over the past decade. Those in favor of a tan believed that they looked better. Subsequently, as these children became young adults, their attitudes toward a tan appearing more attractive increased significantly.^11^ This desire to obtain a tan has led to the expansion of artificial tanning methods in most Western countries.^5^

With such variation in implementation of regulations worldwide, the purpose of our survey was intended to uncover how regulations differ between solariums conducting business in 3 countries, each with varying degrees of legislation. Australia and New Zealand are 2 countries with the highest incidence of melanoma in the world and were therefore selected as a comparison to UK practices.^12^

## MATERIALS AND METHODS

A questionnaire was developed specifically for this survey. It has not been validated but based on the International Commission on Non-Ionizing Radiation Protection and WHO recommendations. A female-simulated customer visited each of the solariums face-to-face and pretended to be a prospective customer, asking all questions that were on the proforma, and completing this after departure from the solarium. The prospective customer did not use a sunbed in any of the individual institutions. The same simulated customer was used in every tanning salon. All solariums chosen were randomly allocated from 3 major cities in the United Kingdom (London, Bristol, Manchester) and New Zealand (Wellington, Christchurch, and Rotorua) and 4 major cities in Australia (Cairns, Brisbane, Sydney, and Townsville). Twenty-one solariums were selected from each country to make a total of 63 solariums visited. We ensured that franchise businesses were not visited more than once to avoid any bias in their training regulations. All spray-tanning and coin-operated solariums were excluded from the directory list.

We asked a set of questions that were relevant to WHO recommendations. The standard questions asked included the following:

1. Do you carry out a skin assessment prior to sunbed use?
2. Can you clarify the potential side effects, given the recent public reports, in particular regarding skin cancer risks?
3. Do I have to sign a consent form?
4. Do you supply eye protection?
5. How long do I have to wait before booking my next session?
6. How long are your average sessions?

## RESULTS

All of the solariums visited were compliant with the study, with 63 parlors answering all the questions.

Overall percentage of total solariums within a country that followed the regulations reveals an overall low proportion of conformity (Table 1). The standard set by our sample of solariums was highest in Australia (41%) and the United Kingdom (39%), followed by New Zealand (20%).

The regulation least satisfied was the consent form. Fourteen percent of solariums in New Zealand requested completion of a consent form, compared to 100% of solariums in both Australia and the United Kingdom. Few solariums in New Zealand adequately informed customers of the potential adverse side effects of sunbed use including skin cancer risks.

**Figure 1:**
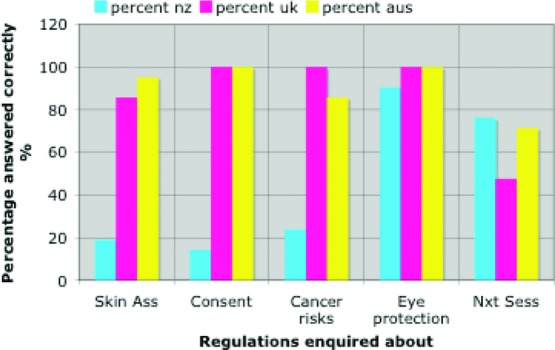
Percentage of 21 solariums conforming to a sample of WHO recommendations in New Zealand, the United Kingdom, and Australia.

Figure 1 illustrates variable tested for each country. The variable best carried out by individual solariums in all 3 countries was the salon's ability to provide eye protection for customers.

## DISCUSSION

Despite our small sample size, our data have shown several differences in the way sunbed parlors conduct their business, with a degree of variation in each country. A plausible reason for these differences is the effect of differences in solarium regulations worldwide.^13^ Previous studies conducted in Australia in which 176 solariums were interviewed found that only 15.9% of those interviewed fulfilled 10 of the 13 recommendations by the WHO.^13^ Studies have shown little improvement in the maintenance of a safe standard of care since implementation of legislative reform.^14^

Solariums in New Zealand were less likely to comply with regular skin assessments, filling in consent forms and providing explanation of potential cancer risks to customers. It has been suggested that before beginning a tanning course, all sunbed operators should ensure that a consent form is signed and dated on the first visit and kept for at least 2 years in the establishment.^5^

Studies have shown that sunbed use increases melanoma risk if its use begins earlier in life.^4^ The International Agency for Research on Cancer concluded that the causal relationship between UV rays and melanoma was further increased with sunbed exposure before the age of 35 years.^2^ This, therefore, implies that interventions should be heavily targeted toward the young adult population, those with increased vulnerability to the carcinogenic impact of indoor tanning.^11^ There is a need to increase public awareness on the dangers of sunbed use. Education and media campaigning have been implemented with some success. Australia has been running a campaign for sun-related behavior called Sunsmart since 1988. A 15-year-long study shows a general improvement in sun-protective behavior, suggesting that protective behaviors are amenable to change with education.^15^ New Zealand has fewer campaigns compared with Australia, with claims that their adolescent population lacks sun protection knowledge and understanding of the threat UV light poses.^16^ One study conducted in New Zealand found that young individuals were unsure about the risks posed by artificial sunbeds.^17^ This gap in knowledge is not only unique to young New Zealanders with similar outcomes in the United Kingdom, where even fewer campaigns exist. Recent suggestions imply that tanning be considered as a form of addiction, with users exhibiting a degree of physiological dependence.^18^

Despite the common misbelief that sunbeds provide a “safer” method of tanning, the emission spectrum at times has been found to be 10 to 15 times greater than the midday sun in the Mediterranean.^3^ In this study, we measured average length of sessions as one of our variables. Tanning bed sunlamps have changed over time in the frequency of wavelengths emitted. Therefore, as a measure we suggest that this should not be interpreted as absolute, since the emission spectrum was unknown.

Our survey was limited in that it involved an interview that required yes and no answers. It was difficult to obtain proof for these claims. However, other studies have suggested that utilizing this method of visiting sunbed parlors is an effective way of assessing current legislation in Australia, as well as providing the media attention to further promote a downward trend in sunbed usage.^19^ To our knowledge, no other surveys have compared solarium use in different countries. Most studies have attempted to evaluate attitudes and motivations of users, with little inquiry into those entrusted as “service providers.” The continued growth in the solarium industry, coupled with the emergence of evidence suggesting adverse health effects linked to sunbed usage, highlights the need for public education. Our findings by no means conclude how each country conducts and conforms to regulations. They are a sample of solariums in each country that provide an insight into how solariums in each country conform to regulations. To provide data for comparison of each country, greater sample size and a more robust study design is needed. Despite this, they do reflect the improvements that can be made at service provider level to protect users. We have demonstrated the variation that exists between solariums worldwide and emphasizes the need for larger-scale studies. A more uniform method of service provision is suggested.

## Figures and Tables

**Table 1:** Overall percentage of 21 solariums within each country conforming to survey regulations

Country	Percentage
New Zealand	20
The United Kingdom	39
Australia	41
